# FGFR3-TACC3 fusion gene promotes glioblastoma malignant progression through the activation of STAT3 signaling pathway

**DOI:** 10.3389/fonc.2025.1560008

**Published:** 2025-04-08

**Authors:** Yiming Li, Jianshen Liang, Xiude Ren, Jiahe Guo, Xisen Wang, Xuya Wang, Shengping Yu, Tao Li, Xuejun Yang

**Affiliations:** ^1^ Department of Neurosurgery, Tianjin Medical University General Hospital, Tianjin, China; ^2^ Laboratory of Neuro-Oncology, Tianjin Medical University General Hospital, Tianjin, China; ^3^ Department of Neurosurgery, Beijing Tsinghua Changgung Hospital, School of Clinical Medicine, Tsinghua Medicine, Tsinghua University, Beijing, China

**Keywords:** FGFR3-TACC3 fusion gene, glioma, malignant progression, invasion, migration, STAT3 signaling pathway

## Abstract

**Objective:**

The Fibroblast growth factor receptors 3-transforming acidic coiled-coil-containing protein 3 (FGFR3-TACC3, F3-T3) oncogenic fusion gene, identified in malignant tumors such as gliomas and bladder cancer, has been particularly noted in recurrent gliomas where it is considered to drive malignant progression, thus presenting itself as a viable therapeutic target. However, the precise mechanism by which F3-T3 facilitates the malignant progression of glioma is not fully understood.

**Methods:**

Correction analysis of STAT3 and FGFR3 with major glioma mutation types and pan-cancer analysis was conducted using The Cancer Genome Atlas (TCGA) database. A series of phenotypic experiments, including CCK-8, EdU, colony-formation assay, wound healing assay, and transwell assay were conducted to detect the effects of F3-T3 on proliferation, invasion, and migration of glioma cells. The association between F3-T3 and epithelial-mesenchymal transition (EMT) was investigated through enrichment analysis of the E-MTAB-6037 gene chip database and confirmed by western blot. The underling mechanism were further inferred and validated through RNA sequencing, E-MTAB-6037 gene chip data, and western blot. The relationship between p-STAT3 expression and the WHO grade of glioma was evaluated using immunohistochemistry (IHC) and tissue microarray analysis. Furthermore, the results of vivo experiments and IHC has confirmed the impact of F3-T3 on glioma malignant progression and activation of the STAT3 signaling pathway.

**Results:**

The experimental results from this study indicate that F3-T3 accelerates the epithelial-mesenchymal transition (EMT) process in glioma cells, thereby promoting their proliferation, invasion, and migration capabilities. Mechanistically, it was determined through RNA sequencing that the signal transducer and activator of transcription 3 (STAT3) signaling pathway is crucial for the malignant progression of F3-T3. This finding was further supported through follow-up experiments conducted after STAT3 knockdown. The role of the STAT3 pathway in gliomas was also reinforced through bioinformatic analysis and immunohistochemistry (IHC) on tissue microarrays (TMA). Further *in vivo* experiments corroborated the role of F3-T3 in enhancing glioma growth and progression.

**Conclusion:**

F3-T3 facilitates the proliferation, invasion, migration and EMT of glioma cells, thereby promoting their malignant progression through STAT3 signaling activation. These findings highlight its potential as a therapeutic target for glioma treatment.

## 1 Introduction

Gliomas, particularly glioblastoma (GBM), which is categorized as WHO grade 4 and represents the most frequent type of primary malignant brain tumor. Based on epidemiological data, GBM constitutes approximately 50% of all adult human brain malignancies, marked by their high incidence, high mortality rates, and poor prognosis (1, 2). For decades, the primary treatment modalities for gliomas have included surgical resection, followed by chemoradiotherapy and sequential chemotherapy (3). Advances in molecular diagnostics have improved our ability to refine glioma diagnoses and assess prognosis. Nevertheless, prolonged use of temozolomide (TMZ) often leads to the happening of drug resistance and further malignant evolution of the tumors. Recently, the focus has shifted towards exploring immunotherapy and molecular targeted therapies, which continue to evolve (4, 5). Despite numerous genes being identified as factors in GBM malignancy, a thorough understanding of their roles in disease pathogenesis still demands significant research and breakthrough discoveries (6–9).

The development of tumors is frequently accompanied by chromosomal translocations and deletions, which lead to genomic rearrangements including the breakdown and rejoining of DNA. Gene fusion always result in the production of expression products of fusion proteins with abnormal sequence or function (10). These proteins can alter gene promoters, leading to the overexpression of certain proteins and impacting the regulation of tumor suppressor genes, thereby influencing oncogenic processes (11). Several fusion genes have become hallmarks of specific cancers. Chronic myeloid leukemia is identified by the presence of BCR-ABL fusion gene, which arises from the translocation of chromosomes 9 and 22 (12). This fusion gene can concurrently activate PI3K and MAPK signaling pathways and their downstream effectors, thereby promoting cell proliferation and enhancing resistance to apoptosis (13, 14). FGFR fusions constitute approximately 10% of all FGFR mutations observed in malignant tumors and have been identified in a variety of cancers, including cholangiocarcinoma, breast cancer, bladder cancer, and glioblastoma (15–18). The F3-T3 fusion gene, results from the partial fusion of FGFR3 and TACC3, retains the kinase activity of FGFR3 and is capable of activating signaling clusters (19). F3-T3 has been identified in both GBM and urothelial bladder cancer (20, 21). F3-T3 confirmed to be associated with chemotherapy resistance and more aggressive of glioma (9, 21). Therefore, elucidating the molecular mechanisms by which F3-T3 drives the malignant progression of glioma cells can provide theoretical insights and practical support for the development of therapeutic approaches in glioma patients harboring the F3-T3 fusion gene.

The STAT3 signaling pathway, capable of being activated by various of cytokines, has been linked to proliferation, invasion, migration, and EMT in numerous malignancies (22–26). However, the specific relationship between the F3-T3 fusion gene and STAT3 signaling has not yet been systematically and comprehensively analyzed, indicating a need for more detailed research. In this study, we first examined the association between the F3-T3 and the malignant progression of gliomas. To assess the potential role of the F3-T3 fusion gene in glioma patients, we analyzed the FGFR3 expression and the survival of patients based on TCGA database. Then, we conducted Gene Set Enrichment Analysis (GSEA) of GSE42401 database to reflect the role of F3-T3 in glioma cells. Additionally, we investigated the effects of F3-T3 on the proliferation, migration, and invasion of GBM cells. Our data suggest that F3-T3 may drive the malignant progression of GBM cells through the activation of STAT3 signaling. This hypothesis was supported by RNA sequencing data, validated by gene chip analysis (E-MTAB-6037), and further experimental findings. Notably, tissue microarray (TMA) and immunohistochemistry (IHC) analysis of phosphorylated STAT3 (p-STAT3) underscored the importance of STAT3 signaling in glioma. Furthermore, in results of vivo experiments showed that the F3-T3 group exhibited higher levels of phosphorylated FGFR3 (p-FGFR), p-STAT3, and Ki-67 compared to the empty vector and F3-T3 with STAT3 knockdown (sh-STAT3+F3-T3) groups. Overall, our results indicate that the F3-T3 fusion gene promotes the malignant progression of GBM cells through STAT3 signaling activation, presenting it as an important therapeutic target for GBM treatment.

## 2 Methods

### 2.1 Bioinformatic analysis and RNA sequencing

The E-MTAB-6037 chip data and GSE42401 were obtained from previous researchers, detailed descriptions of those database are provided in the **Supplementary Methods** (27–29). The methodologies used have been described previously (24). In **Supplementary Methods**, we described the main process of bioinformatic analysis based on TCGA database.

The RNA sequencing process conducted was as following: Total RNA was extracted from U251MG cells containing either F3-T3 or an empty vector using TRIzol. Following RNA collection, reverse transcription, library preparation, and sequencing were processed by Novogene Zhiyuan Technology Co., Ltd., (Beijing, China). Differential expression analysis was carried out using the “DESeq2” package in R, and pathway analysis was obtained with the Kyoto Encyclopedia of Genes and Genomes (KEGG). The entire RNA sequencing process was replicated in triplicate.

### 2.2 Cell culture

Human glioma cell U251MG and U87MG were cultured in DMEM medium supplemented with 10% fetal bovine serum (FBS). Detailed information exhibited in **Supplementary Methods**.

### 2.3 Lentivirus and siRNA transfection

Lentiviruses were obtained from Genechem (China). STAT3 siRNA, obtained from Genepharma (China). The sequences of lentivirus, internal reference, and siRNA have been provided in **Supplementary Methods**.

### 2.4 Cell Counting Kit-8 and 5-ethynyl-2’-deoxyuridine assays

The detailed protocol of CCK-8 has been described previously (24). The EdU labeling procedure was performed following the instruction book provided by Elabscience, as described previously (30). The main protocol of CCK-8 and EdU assay were described in **Supplementary Methods**.

### 2.5 Transwell assay

The details of transwell assay (including migration and invasion assay) were exhibited in **Supplementary Methods**.

### 2.6 Colony formation assay

The main process of colony formation assay could be seen in **Supplementary Methods**.

### 2.7 Cell wound healing assay

The experiment was performed as **Supplementary Methods** described.

### 2.8 Western blotting

The main procedure used for western blotting has been described previously (31). The primary antibodies utilized for the western blots are shown in **Supplementary Methods**.

### 2.9 Tissue microarray

TMA samples were obtained from clinical specimens of glioma patients in our department. The main process of tissue collection and specimen information were listed in **Supplementary Methods**.

### 2.10 Xenograft mouse model

Previous studies have outlined the procedure for establishment of mouse xenograft models (24, 31). The main protocol of animal experiments was indicated in **Supplementary Methods**. The animal experiments have received approval from the Ethical Committee of Tianjin Medical University General Hospital.

### 2.11 H&E staining and IHC

The brains containing tumor from nude mice were embedded, and H&E staining were performed as previously described (31). **Supplementary Methods** have described the specific methods. The protocol for subsequent analysis was based on methods described in an earlier study (24).

### 2.12 Statistical analysis

The primary methods and software for statistics were described in **Supplementary Methods**.

## 3 Results

### 3.1 F3-T3 promoted the malignant progression of glioma cells

**Figure 1:**
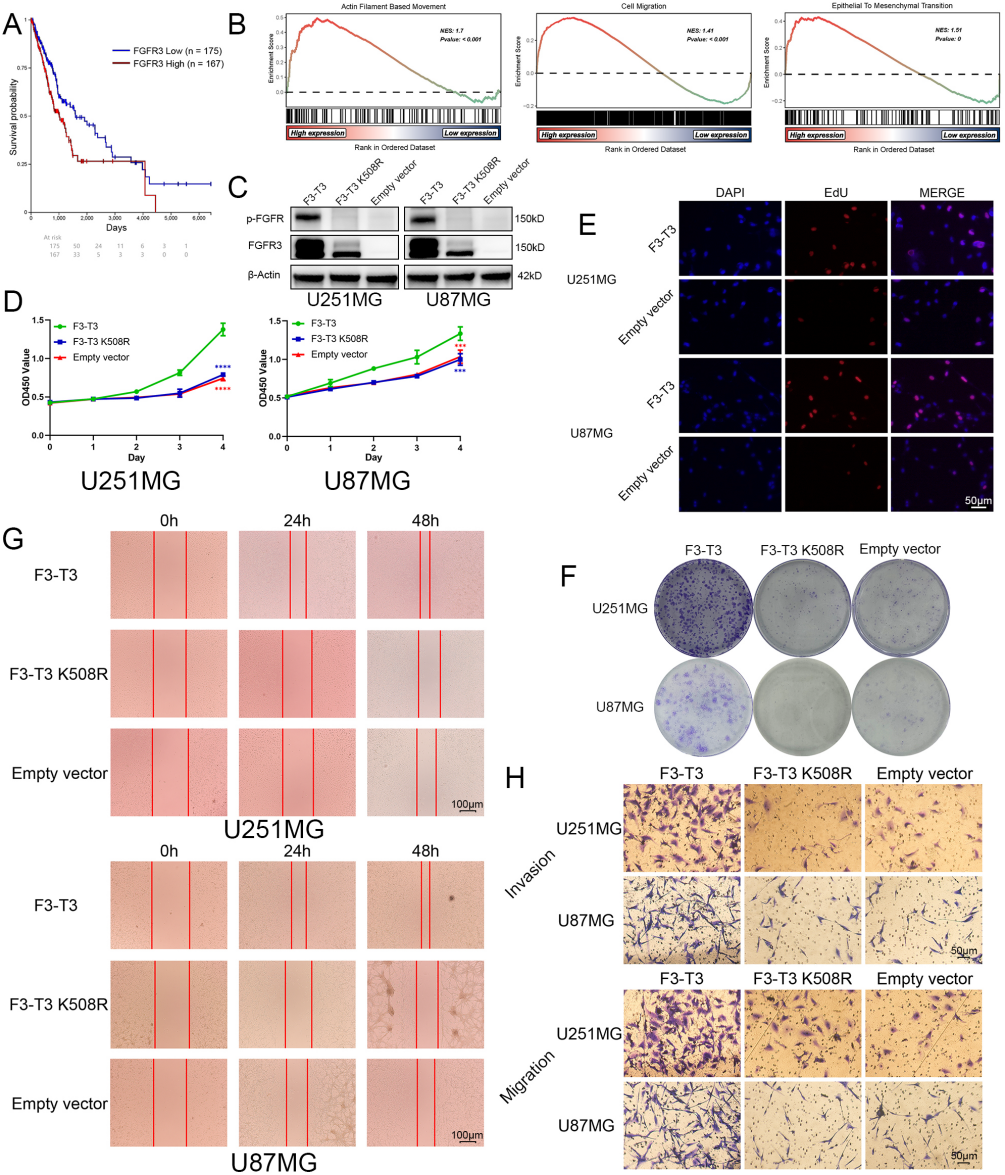
F3-T3 significantly promoted the proliferation, invasion, and migration of glioma cells. **(A)** Correlation of FGFR3 expression with prognosis in glioma patients from the TCGA database: The Kaplan-Meier plotter showed Glioma patients with higher FGFR3 expression reached poorer prognosis than those expressed lower. **(B)** GSEA results of F3-T3 and empty vector based on the GSE42401 database. The GSEA results showed F3-T3 glioma cells enriched in several key signaling pathways that closely correlated with cancer. **(C)** Western blot analysis showing expression levels of p-FGFR and FGFR3 in cells transfected with F3-T3, F3-T3 K508R, and empty vector. **(D)** CCK-8 assay results indicating enhanced proliferation of U87MG and U251MG cells expressing F3-T3 compared to those with F3-T3 K508R mutant and empty vector. **(E)** EdU labeling assay demonstrating increased proliferation in U87MG and U251MG cells due to F3-T3 expression. **(F)** Colony formation assay results showing higher colony formation efficiency in glioma cells expressing F3-T3 compared to control groups. **(G)** Wound healing assay results revealing increased invasion capability in glioma cells with F3-T3 expression compared to cells with F3-T3 K508R mutant and empty vector. **(H)** Transwell assays for invasion and migration showing that F3-T3 can significantly enhance these abilities in glioma cells compared to F3-T3 K508R and empty vector. (***p<0.001, ****p<0.00001).

Initially, we conducted a comprehensive bioinformatics analysis of FGFR3 and F3-T3. Firstly, we included the Kaplan-Meier survival curves for glioma patients with high and low FGFR3 expression from TCGA database, we can observe that glioma patients with higher FGFR3 expression always have poor prognosis than those with lower FGFR3 expression (**Figure 1A**) (*p* = 0.008). Then, we conducted differential expressed genes (DEGs) analyzed based on GSE42401 database (29). After DEGs were analyzed, we carried out GSEA for several key signalings that closely correlated with malignant progression of cancer. As illustrated from GSEA results, we could find that Actin Filament-Based Movement, Cell Migration, and Epithelial-to-Mesenchymal Transition was highly activated in F3-T3 GBM cells (**Figure 1B**) (*p* < 0.05). Besides, we transfected U87MG and U251MG glioma cells with F3-T3, its kinase-inactive form (F3-T3 K508R), and an empty vector using lentiviral vectors. Western blot results confirmed successful transfection, indicating significantly higher expression levels of p-FGFR and FGFR3 in the F3-T3 group compared to both the F3-T3 K508R and empty vector groups (**Figure 1C**). Additionally, FGFR3 expression in the F3-T3 K508R group was higher than the expression in empty vector group (**Figure 1C**). We further assessed the impact of F3-T3 on cell proliferation and colony formation through CCK-8 assays, colony-formation assays, and EdU fluorescence labeling. CCK-8 results showed that cells harboring F3-T3 had a higher proliferative capacity than those in the F3-T3 K508R and empty vector groups (**Figure 1D**), this finding was supported by EdU assay results (**Figure 1E**). Moreover, the results of colony formation assay revealed stronger colony-forming abilities in the F3-T3 group compared to the others (**Figure 1F**, **Supplementary Figure S1**). These experiments demonstrate that F3-T3 enhances glioma cell proliferation and colony formation. To determine the effects of F3-T3 on cell migration and invasion, we conducted wound healing and transwell assays. The results showed that F3-T3 glioma cells displayed increased invasive and migratory capabilities (wound healing assay: **Figure 1G**, **Supplementary Figure S2**, transwell assay: **Figure 1H**, **Supplementary Figure S3**). Together, these findings indicate that the F3-T3 plays a significant role in driving the malignant progression of glioma cells.

### 3.2 Detection of EMT and STAT3 signaling in glioma cells harboring F3-T3

**Figure 2:**
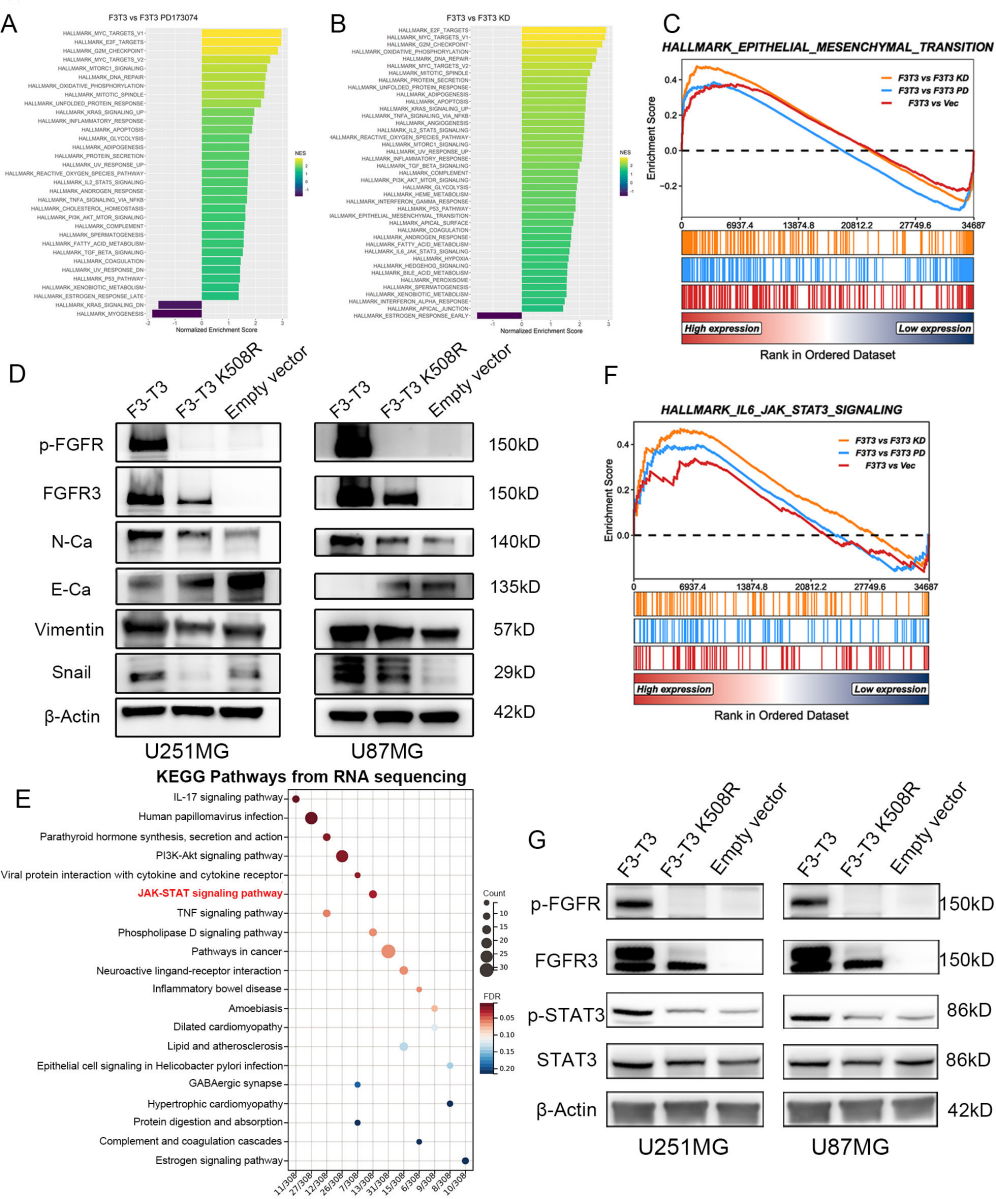
F3-T3 was highly correlated with the EMT process and STAT3 signaling in gliomas. **(A, B)** GSEA and HALLMARK pathway analysis performed on the E-MTAB-6037 gene chip database to identify enriched pathways in the comparison groups. **(C)** GSEA results from the E-MTAB-6037 gene chip data demonstrating enrichment of F3-T3 associated genes in the EMT process, with an adjusted p-value < 0.05. Specific Normalized Enrichment Scores (NES) and adjusted p-values are detailed in **Supplementary Figure S4**. **(D)** Western blot analysis showing differential expression of EMT markers among three groups in U87MG and U251MG glioma cells. Increased expression of N-Cadherin, Vimentin, and Snail was observed in the F3-T3 group, whereas E-Cadherin expression was decreased, compared to the other two groups. **(E)** The top 20 KEGG enrichment pathways for differentially expressed genes (DEGs) from RNA sequencing comparing F3-T3 to empty vector in U251MG glioma cells. A KEGG bubble plot highlights the enrichment of the JAK-STAT signaling pathway, with an adjusted p-value of 0.025309 (detailed pathway table shown in **Supplementary Table S1**). **(F)** GSEA of E-MTAB-6037 gene chip data indicating that genes associated with the F3-T3 fusion are enriched in the IL6-JAK-STAT3 signaling pathway (*p* < 0.05). **(G)** Western blot analysis of U251MG and U87MG glioma cells comparing the expression levels of key markers in the STAT3 signaling pathway. Elevated expression of phosphorylated STAT3 (p-STAT3) was noted in the F3-T3 group, with no significant differences in total STAT3 expression across groups.

We analyzed RNA-sequencing data from the E-MTAB-6037 gene chip to elucidate the role of F3-T3 (28). Differential gene analysis and subsequent GSEA identified potential association of F3-T3 with various cancer-related pathways, including the interleukin 6-janus kinase-STAT3 (IL6-JAK-STAT3) pathway, indicating its role in the malignant progression of glioma cells (**Figures 2A, B**). The results of the gene chip data indicated the significant enrichment of EMT in the F3-T3 group (*p* < 0.05, **Figure 2C**, specific figures listed in **Supplementary Figure S4**). Furthermore, western blotting was used to assess the primary EMT markers, revealing elevated expression of N-Cadherin, Vimentin, and Snail in the F3-T3 group, while E-Cadherin was reduced, aligning with the bioinformatics results (**Figures 2C, D**). To uncover the underlying mechanisms by which F3-T3 induces EMT, we performed RNA sequencing on U251MG cells harboring F3-T3 and an empty vector. The KEGG enrichment analysis was conducted after differential genes were examined. In **Figure 2E**, we could observe the JAK-STAT signaling was enriched (*p* value = 0.001092, the top 20 KEGG enrichment signaling was listed in **Supplementary Table S1**). Further analysis of the IL6-JAK-STAT3 signaling pathway using the E-MTAB-6037 gene chip database corroborated our RNA sequencing results (*p* < 0.05, **Figure 2F**) (28). Subsequent western blot results confirmed a significant increase in p-STAT3 expression in F3-T3 glioma cells, while overall STAT3 levels remained constant, supporting the above results of the bioinformatic analyses (**Figure 2G**). Collectively, these findings indicate that F3-T3 activates STAT3 signaling, thereby enhancing the malignant progression of glioma cells.

### 3.3 STAT3 is related to the EMT, WHO grades, and poor prognosis of glioma

**Figure 3:**
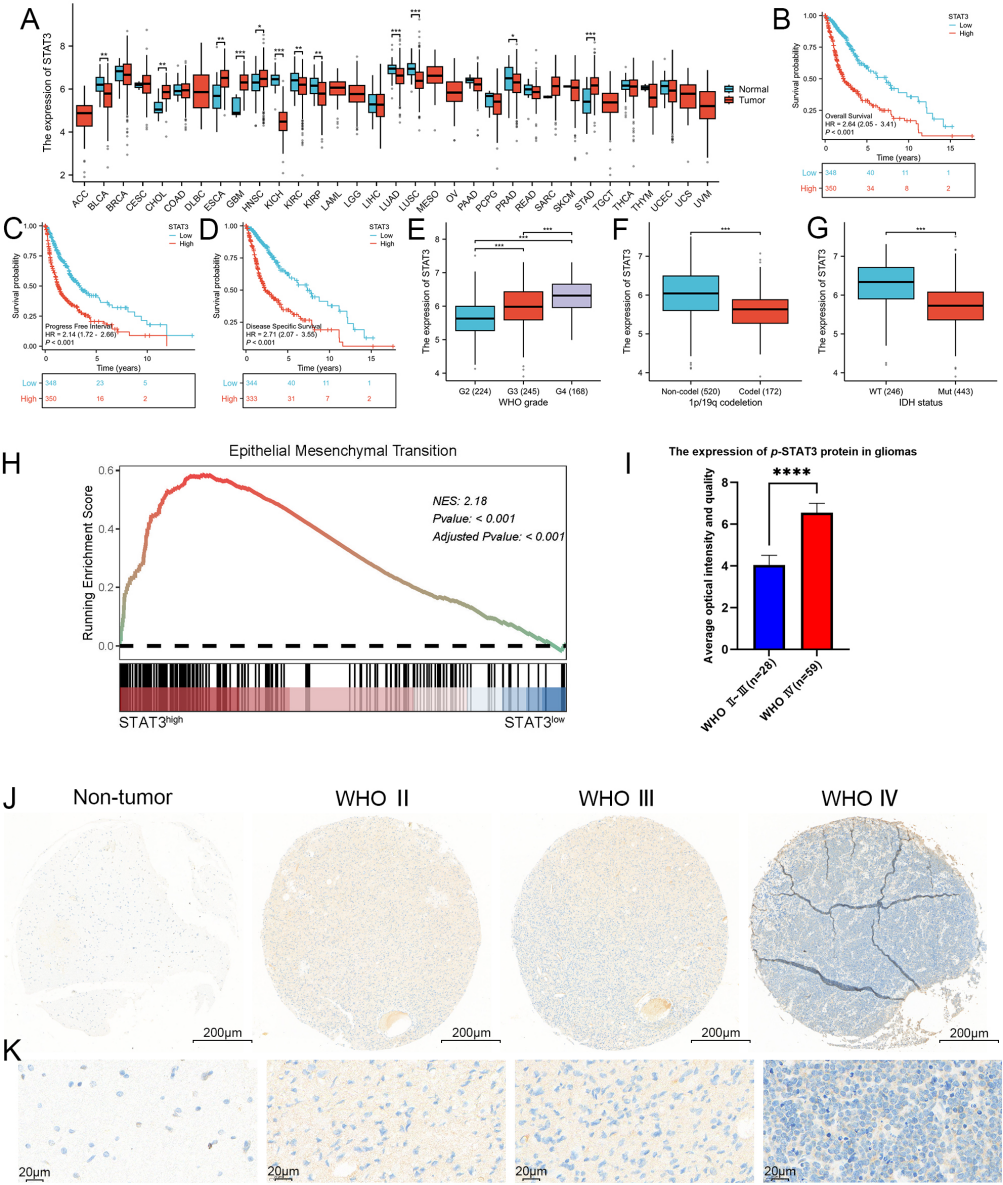
STAT3 was closely associated with the WHO grade, IDH wild-type status, 1p/19q chromosome co-deletion and worse prognosis, as well as being closely linked to EMT. **(A)** Expression profile of STAT3 across 33 different cancer types and their corresponding normal tissues, as shown in the TCGA database. Our analysis revealed that in 9 of the 33 malignant tumor types, the expression profiles did not match those of their corresponding normal tissues, these malignant tumors including lymphoid neoplasm diffuse large B-cell lymphoma (DLBC), adrenocortical carcinoma (AAC), brain lower grade glioma (LGG), mesothelioma (MESO), acute myeloid leukemia (LAML), uveal melanoma (UVM), testicular germ cell tumors (TCGT), ovarian serous cystadenocarcinoma (OV), and uterine carcinosarcoma (UCS). Among the remaining 24 cancer types, 12 exhibited statistically significant differences in STAT3 expression levels between tumor and normal tissues. These cancers included bladder and urothelial carcinoma (BLCA), esophageal carcinoma (ESCA), cholangiocarcinoma (CHOL), glioblastoma (GBM), kidney renal clear cell carcinoma (KIRC), head and neck squamous cell carcinoma (HNSC), kidney chromophobe (KICH), lung adenocarcinoma (LUAD), kidney renal papillary cell carcinoma (KIRP), stomach adenocarcinoma (STAD), lung squamous cell carcinoma (LUSC), and prostate adenocarcinoma (PRAD). In contrast, the other 12 cancers did not show statistically significant differences. Specifically, five cancer types demonstrated elevated STAT3 expression in tumor tissues, including cholangiocarcinoma (CHOL), glioblastoma (GBM), esophageal carcinoma (ESCA), head and neck squamous cell carcinoma (HNSC), and stomach adenocarcinoma (STAD), while seven showed higher expression in normal tissues, including bladder and urothelial carcinoma (BLCA), kidney renal clear cell carcinoma (KIRC), kidney chromophobe (KICH), lung squamous cell carcinoma (LUSC), kidney renal papillary cell carcinoma (KIRP), lung adenocarcinoma (LUAD), and prostate adenocarcinoma (PRAD). Notably, GBM exhibited a marked difference in STAT3 expression between tumor and normal tissues (*p* < 0.001). **(B–D)** Kaplan-Meier survival curves analyzing the overall survival, progression-free interval, and disease-specific survival of glioma patients categorized by high versus low STAT3 expression. The curves indicate that higher STAT3 expression was associated with poorer prognosis in glioma patients. **(E–G)** Correlation analysis between STAT3 expression levels and clinical parameters in glioma, including WHO grades, 1p/19q chromosome co-deletion status, and IDH mutation status, utilizing the TCGA database. The analysis results shows that STAT3 expression was significantly linked to these clinical indicators. **(H)** GSEA analysis revealed a strong correlation between STAT3 expression and the EMT process, with a NES of 2.1825 and an adjusted p-value < 0.001. **(I, J)** Analysis of p-STAT3 expression in GBM, demonstrating significantly higher levels compared to lower-grade gliomas. **(K)** Enlarged histological image results of **(J)**. (*p<0.05, **p<0.01, ***p<0.001, ****p<0.00001).

To explore the role of the STAT3 signaling pathway in glioma, we analyzed both the expression levels of the STAT3 gene and its corresponding protein. Initially, we analyzed data of the TCGA database to assess the expression profiles of STAT3 across various malignant tumors, comparing these with non-pathological tissue expression values from the GTEx database (**Figure 3A**). We found that in 9 out of 33 malignant tumor types, the expression profiles did not match those of their corresponding normal tissues. Among the other 24 cancers, 12 showed statistically significant differences in STAT3 expression between the tumor and normal tissues. Conversely, the remaining 12 cancers did not show a statistically significant difference. Specifically, five cancers demonstrated higher STAT3 expression in tumor tissues. Meanwhile, seven cancers showed higher expression in normal tissues. Notably, in GBM, we could observe a markable difference in STAT3 expression between tumor and normal tissue (detailed results were exhibited in **Figure 3A**, p < 0.001).

A comprehensive survival analysis using the TCGA database revealed that glioma patients with elevated STAT3 expression had reduced overall survival, progress free interval, and disease specific survival compared to those with expressed lower levels of STAT3 (*p* < 0.001, **Figures 3B–D**). Additionally, we assessed WHO classification, chromosome 1p/19q status, and IDH status from the TCGA database and categorized them accordingly. According to the guidelines of the WHO 5th edition, a positive correlation was observed between STAT3 expression and WHO grade (*p* < 0.001) (**Figure 3E**). In **Figure 3F**, it was observed that 1p/19q non-codeletions were associated with higher STAT3 expression (*p* < 0.001). Furthermore, glioma patients with IDH wild-type status exhibited higher STAT3 expression compared to those with IDH mutations (Mut) (*p* < 0.001), (**Figure 3G**). STAT3 signaling, which is frequently activated in malignant tumors, correlates strongly with several malignancy traits. The process of EMT can endow cells with enhanced invasive and migratory capabilities, stem cell-like characteristics, resistance to apoptosis, and immunosuppressive effects. EMT is critical not only in physiological contexts such as embryonic development and wound healing but also in the metastasis of tumors. This involves a transition from epithelial cell markers to an increase in mesenchymal cell markers. To further investigate the association between STAT3 signaling and the EMT process in greater depth, we examined DEGs related to STAT3 in GBM from the TCGA database. Importantly, the GSEA results indicated a close association between STAT3 and EMT (NES = 2.1825, *p* < 0.001) (**Figure 3H**).

To investigate the expression of p-STAT3 protein in glioma tissues within a clinical setting, we utilized IHC on TMAs sourced from our department. The analysis indicated that p-STAT3 was predominantly expressed in glioma tissues (**Supplementary Figure S5**). Additionally, a systematic evaluation of specimens across different WHO grades showed that p-STAT3 expression was significantly higher in WHO grade IV gliomas compared to WHO grades II and III (**Figures 3I, J**). Furthermore, p-STAT3 levels were notably elevated in the central areas of tumors relative to peritumoral tissues. The IHC staining results comparing different WHO grades of glioma are depicted in **Figures 3J** and **K**.

### 3.4 F3-T3 promotes the malignant progression of glioma through the activation of STAT3 signaling

**Figure 4:**
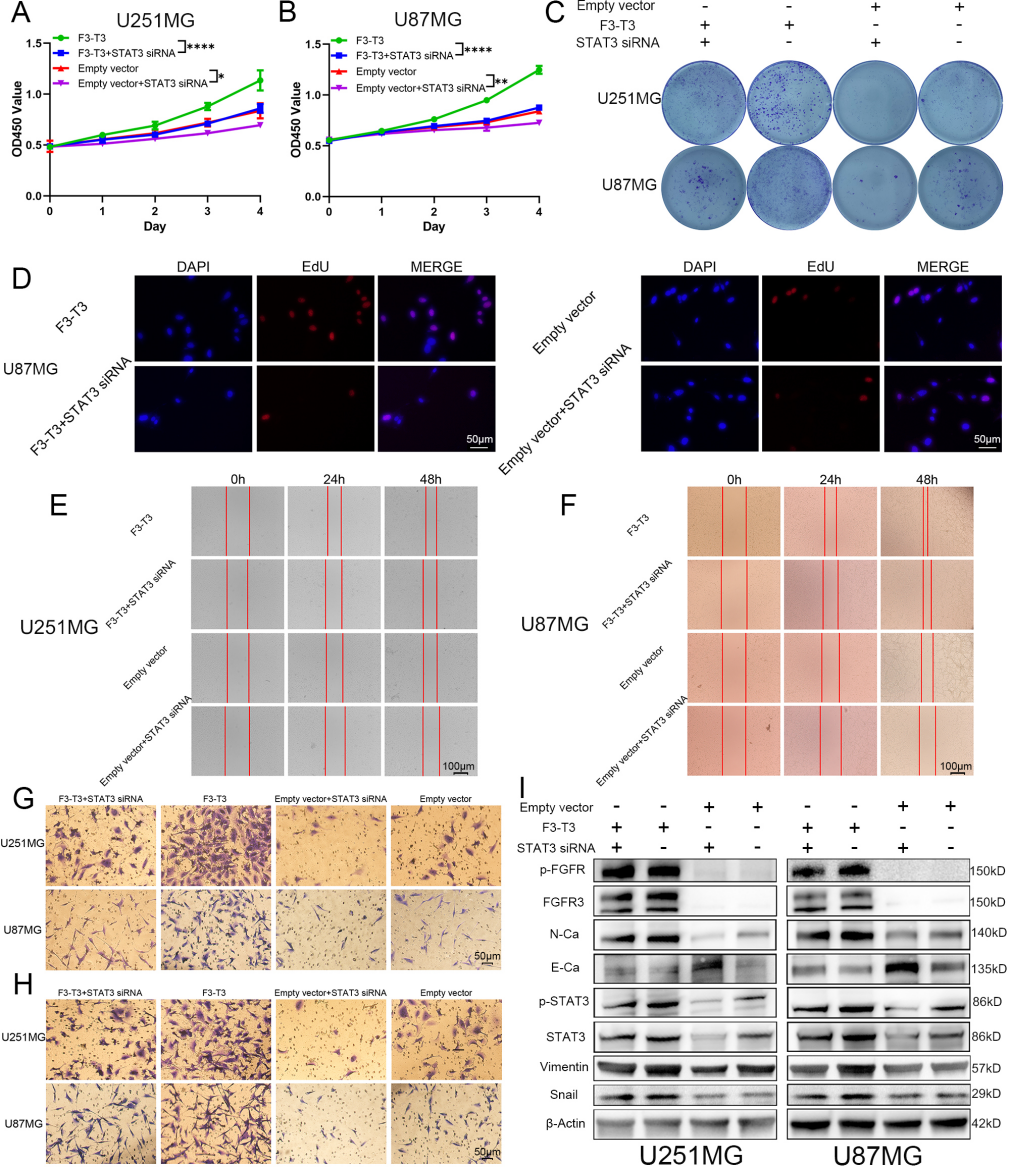
F3-T3 was found to mediate the proliferation, invasion, and migration of glioma cells through the activation of the STAT3 signaling pathway. **(A, B)** CCK-8 assays demonstrating reduced proliferation in U87MG and U251MG glioma cells following STAT3 knockdown. **(C)** Colony formation assays showing a significant decrease in colony formation efficiency in glioma cells post-STAT3 knockdown. **(C)** EdU labeling assay showed a reduction in the proliferative capacity of U87MG cells, both in those expressing F3-T3 and those carrying the empty vector, after the knockdown of STAT3. **(E, F)** Wound healing assays illustrate that STAT3 knockdown leads to decreased invasion capabilities in glioma cells with both F3-T3 and empty vector. **(G, H)** Transwell assays showing that knockdown of STAT3 reduces both invasion **(G)** and migration **(H)** abilities in glioma cells expressing either F3-T3 or empty vector. **(I)** Western blot analysis revealing changes in the expression levels of p-FGFR, FGFR3, E-Cadherin (E-Ca), N-Cadherin (N-Ca), Vimentin, and Snail in four distinct groups of glioma cells. Compared with untreated F3-T3 or empty vector glioma cells, STAT3 knockdown significantly decreases the expression of N-Cadherin, Vimentin, and Snail, while markedly increasing E-Cadherin expression. (*p<0.05, **p<0.01, ****p<0.00001).

Our analysis of data from TCGA database revealed a strong association between STAT3 signaling and the EMT process in GBM. Integrating KEGG enrichment analysis from RNA sequencing of F3-T3 glioma cells with GSEA results from the E-MTAB-6037 gene chip database, we suggest that F3-T3 enhances the EMT process in glioma through the activation of STAT3 signaling. To test this hypothesis, we conducted validation experiments using siRNA to knock down STAT3 and assessed its effects on various phenotypes. In the CCK-8 assay, the proliferation induced by F3-T3 in glioma cells was diminished following STAT3 knockdown (**Figures 4A, B**). The colony formation assay showed a distinct decrease in both proliferation and colony-forming ability of glioma cells post-STAT3 knockdown (**Figure 4C**). These observations were supported by the EdU assay, which confirmed the results from the CCK-8 and colony formation assays (**Figure 4D**, U251MG results are shown in **Supplementary Figure S6**). Besides, we evaluated the effects of STAT3 knockdown on cell invasion and migration through transwell and wound healing assays. The results demonstrated reduced wound healing capacity in both F3-T3 and empty vector groups with STAT3 knockdown (**Figures 4E, F**, **Supplementary Figure S7**). Furthermore, transwell assays indicated that F3-T3 glioma cells with STAT3 knockdown had lower invasion and migration capabilities compared to control cells (invasion: **Figure 4G**, **Supplementary Figure S8**, migration: **Figure 4H**, **Supplementary Figure S9**). Western blot analysis revealed that STAT3 knockdown in F3-T3 cells resulted in decreased expression of N-cadherin, Vimentin, and Snail, with an increase in E-cadherin expression (**Figure 4I**). These findings support the notion that F3-T3 promotes the proliferation, migration, invasion, and EMT of glioma cells through STAT3 signaling activation, thereby facilitating malignant progression.

### 3.5 Silencing of STAT3 impaired the invasion of F3-T3 GBM cells *in vivo*

**Figure 5:**
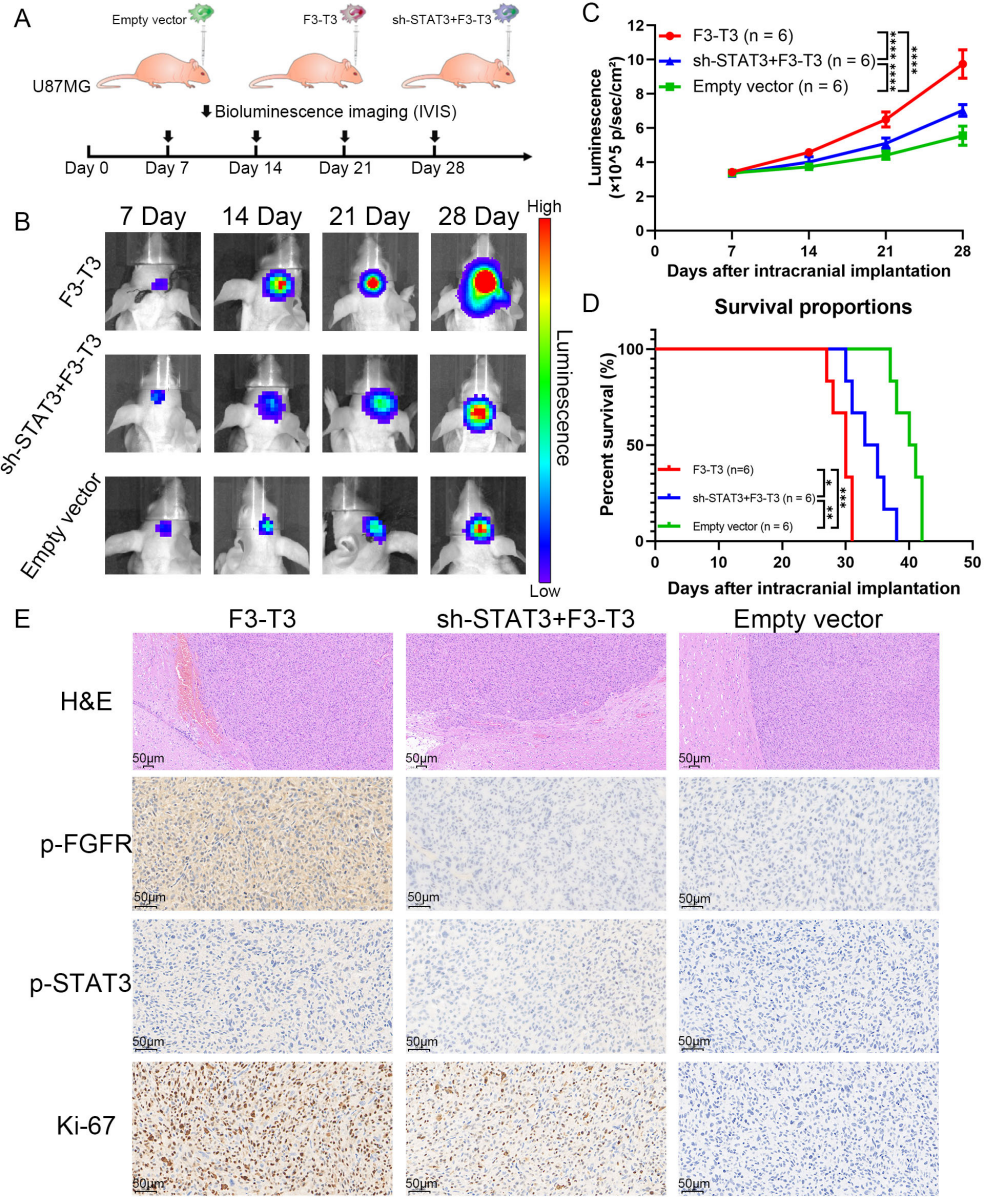
F3-T3 promoted the invasion and progression of GBM cells *in vivo* experiments, while silencing STAT3 impaired this effect **(A)**. A brief overview of the vivo experiment design and group allocation (n = 6 per group). After the implantation of U87MG glioma cells for nude mouse, tumor progression was monitored weekly using bioluminescence imaging. **(B, C)** Bioluminescence images and corresponding line chart depicting the progression of tumor growth over time. Among the three groups in the vivo experiments, the intracranial tumor of F3-T3 group has progressed faster, the knockdown of STAT3 could slow down this process. **(D)** Kaplan-Meier survival curves displaying the survival percentages of the mice across different experimental groups. The results from Kaplan-Meier survival curves confirmed the findings from the bioluminescence results. **(F)** Representative H&E staining images showing the tumor margins in the mouse cerebrum. H&E staining of the F3-T3 group revealed invasive tumor margins and evidence of hemorrhage. IHC staining for phosphorylated FGFR (p-FGFR), phosphorylated STAT3 (p-STAT3), and Ki-67, highlighting the role of the F3-T3 fusion in promoting glioma cell proliferation and tumor progression through the activation of STAT3 signaling. (*p<0.05, **p<0.01, ***p<0.001, ****p<0.00001).

To further confirm the influence of F3-T3 on glioma, we performed *in vivo* experiments using an intracranial xenograft nude mouse model with U87MG glioma cells. After implanting the GBM cells, we monitored tumor growth through bioluminescence imaging conducted weekly from day 7 to day 28. The experimental groups consisted of cells with F3-T3, empty vector, and sh-STAT3+F3-T3, as shown in **Figure 5A**. The bioluminescence results revealed that the F3-T3 expressing glioma cells grew more aggressively, showing significantly higher luminescence levels (**Figures 5B, C**, *p* < 0.05). Importantly, knocking down STAT3 in F3-T3 expressing cells partially reversed these effects. We used the Kaplan-Meier survival curves to evaluate the survival times of the mice, which indicated that mice in the F3-T3 group had a shorter OS in comparation with those in the sh-STAT3+F3-T3 and empty vector groups (*p* < 0.05) (**Figure 5D**). H&E staining of the tumors showed that those from the F3-T3 group had invasive margins with hemorrhagic features, while tumors from the empty vector and sh-STAT3+F3-T3 groups displayed smoother boundaries (**Figure 5E**). Additionally, IHC staining was also performed to detect the expression of p-FGFR, p-STAT3 and Ki-67. The results of IHC staining indicating significantly higher expression levels of p-FGFR, p-STAT3 and Ki-67 in the F3-T3 group compared to the other groups (**Figure 5E**). Furthermore, the knockdown of STAT3 could reverse these effects induced by F3-T3. These results demonstrate that F3-T3 can activates STAT3 signaling and enhance the proliferation, invasion, and migration of glioma cells, aligning with our observations *in vitro*. Overall, these findings support the hypothesis that F3-T3 drives the malignant progression of glioma cells via the activation of STAT3 signaling pathway.

## 4 Discussion

Glioma, the most common primary intracranial malignant tumor, with GBM being its most aggressive form. Patients suffered from GBM usually have a poor prognosis. Although there have been significant advancements in treatments such as tumor treating fields, bevacizumab, and Chimeric Antigen Receptor T-Cell Immunotherapy (CAR-T), the STUPP protocol continues to be the predominant treatment approach for managing glioma (3, 32–36). As treatment progresses, some gliomas may develop more malignant features, including increased invasiveness and enhanced resistance to therapy, which can lead to worsened outcomes. It is crucial that future research aims to identify and target the genes involved in the malignant progression of gliomas.

Gene fusion refers to the combination of partial or complete sequences from two distinct genes, resulting from chromosomal translocations, deletions, or inversions (16, 37–40). These resulting genes are typically called fusion genes. For an extend period, gene fusions have been closely associated with the initiation and progression of various malignant tumors, often acting as primary drivers of cancer development (39). Consequently, with the growing use of next-generation sequencing technology, oncogenic fusion genes have become a focal point of attention and have influenced subsequent therapeutic strategies (41, 42). Targeting oncogenic fusion genes has proven to be a promising therapeutic strategy, and the Food and Drug Administration (FDA) has approved several drugs for treating cancers that possess specific fusion genes (39). However, the oncogenic mechanisms underlying fusion genes are complex and warrant further detailed investigation.

The F3-T3 fusion gene, formed from the combination of the FGFR3 and TACC3 genes, is detected in approximately 3% of gliomas and other malignant tumors, marking it as a notable oncogenic fusion gene (9, 19, 20, 43, 44). Previous studies have explored the metabolic effects, treatment resistance, and tumor-promoting roles of F3-T3 across various cancers (19, 28, 45–49). For instance, Daly C et al. found that F3-T3 can replace the function of EGFR/ERK signaling, thereby enhancing tumor resistance to therapy in head and neck squamous cell carcinoma (48). In gliomas, F3-T3 is associated with increased therapeutic resistance, contributing to a worse prognosis (45). However, the specific oncogenic mechanisms of F3-T3 in glioma are still not fully understood.

In this study, we first illustrated that F3-T3 GBM cells exhibit activation in Actin Filament-Based Movement, Cell Migration, and EMT, which are correlated with malignant progression, based on GSE42401 database (**Figure 1B**) (29).Then, we demonstrated that F3-T3 can facilitate the malignant progression of glioma cells, as evidenced by enhanced proliferation, invasion, and migration (**Figures 1D–H**). The E-MTAB-6037 gene chip database, collected from sequence of three groups of human astrocytes, including human astrocytes containing F3-T3 and those treated with FGFR inhibitor (F3-T3 PD173074), those expressing a kinase-inactive variant (F3-T3 K508M) or an empty vector (vec). The analysis data from this database can better reflect the role of F3-T3 in human astrocytes and even glioma cells. Analysis of the E-MTAB-6037 gene chip data showed that F3-T3 is involved in enriching several signaling pathways such as the cell cycle, G2/M checkpoint, DNA repair, TGF-β, PI3K-AKT, P53, Mitotic Spindle, and EMT (**Figures 2A–C**) (28). The process of EMT is crucial for tumor progression and is closely linked with tumor invasion, migration, and stem cell-like characteristics, which are key attributes of various human malignancies (50, 51). In glioma, enhanced proliferation, invasion, migration, and EMT can accelerate tumor progression and lead to poor patient outcomes (52–55). Further experimental work confirmed that F3-T3 promotes the EMT process in glioma cells, reinforcing its role in driving malignant progression (**Figure 2D**). Additional *in vivo* experiments have corroborated these *in vitro* findings (**Figure 5**).

To explore how F3-T3 promotes the malignant progression of glioma cells, we conducted RNA sequencing. KEGG pathway enrichment analysis using RNA sequencing data indicated significant activation of the JAK-STAT3 signaling in F3-T3 glioma cells (**Figure 2E**). STAT3, initially recognized as a DNA-binding protein, plays a critical role in various cellular processes such as proliferation, survival, differentiation, angiogenesis, and immune responses (25, 56, 57). Activation of STAT3 can be triggered by several cytokines, including IL-6 and IL-10, and certain growth factors (58). These cytokines interact with their receptors to activate JAK, which leads to tyrosine phosphorylation of the receptor and subsequent recruitment and phosphorylation of STAT3 (25, 59). Besides, phosphorylated STAT3 dimerizes and translocates into the nucleus, where it influences cellular processes such as EMT, contributing to tumor initiation and progression (60, 61). In malignant tumors, persistent activation of STAT3 is often linked with adverse clinical outcomes (62). Based on the findings above, we hypothesized that STAT3 is a crucial mediator of the F3-T3-induced malignant progression in glioma. Subsequent experiments and analysis of E-MTAB-6037 gene chip data has confirmed our hypothesis regarding the underlying mechanism (**Figures 2F, G**). GSEA results of GBM data from TCGA database also confirmed a strong correlation between STAT3 signaling and EMT (**Figure 3H**). Notably, after the knockdown of STAT3, we performed various assays that confirmed the reduction in F3-T3-mediated proliferation, migration, invasion, and EMT (**Figure 4**). Further *in vivo* experiments validated these *in vitro* results (**Figure 5**). Overall, our findings demonstrate that F3-T3 enhances the proliferation, invasion, and migration of glioma cells primarily through activating the STAT3 signaling pathway.

To investigate the impact of STAT3 signaling on glioma, we conducted a series of comprehensive analyses. Through systematic analysis of the TCGA database, we detected that the expression of STAT3 correlates with critical indicators of glioma, such as WHO grade, 1p/19q chromosome, and IDH mutation status (**Figures 3E–G**). Furthermore, IHC staining of tissue microarrays showed a significant association between p-STAT3 protein levels and the WHO grade of glioma, underscoring the significance of the STAT3 signaling pathway (**Figures 3I–K**, **Supplementary Figure S5**). In hence, our findings clearly indicate that STAT3 signaling plays a significant role to the progression of glioma.

As a preliminary exploration, this study has several limitations. Firstly, the precise mechanisms and modes of action through which F3-T3 can affect the STAT3 signaling pathway remain to be fully elucidated. Moreover, this study did not include preclinical or clinical assessments of STAT3 inhibition in gliomas driven by F3-T3. Future studies should concentrate on detailed investigations into the mechanisms of F3-T3 and the development of targeted therapies.

In conclusion, this study investigated the primary mechanisms by which F3-T3 influences glioma. We have demonstrated that F3-T3 facilitates the malignant progression of glioma cells via the activation of the STAT3 signaling. Our detailed analysis, utilizing systematic approaches, established that both the expression of STAT3 and the STAT3 signaling activities are strongly associated with higher WHO grades of glioma, the presence of 1p/19q chromosome 1p/19q non-codeletions, and IDH wild-type status. These findings confirm the pivotal role of F3-T3 as a significant oncogenic driver in gliomas. Thus, understanding how F3-T3 contributes to tumor dynamics offers valuable insights into the molecular underpinnings of glioma and highlights its potential as a target for therapeutic intervention. This study underscores the importance of further exploring the role of F3-T3 in glioma progression, which could lead to the development of more effective treatment strategies, potentially improving outcomes for glioma patients with this oncogenic fusion gene.

## Data Availability

The datasets presented in this study can be found in online repositories. The names of the repository/repositories and accession number(s) can be found in the article/**Supplementary Material**.
