# Correction: FGFR3-TACC3 fusion gene promotes glioblastoma malignant progression through the activation of STAT3 signaling pathway

**DOI:** 10.3389/fonc.2025.1637363

**Published:** 2025-07-02

**Authors:** Yiming Li, Jianshen Liang, Xiude Ren, Jiahe Guo, Xisen Wang, Xuya Wang, Shengping Yu, Tao Li, Xuejun Yang

**Affiliations:** ^1^ Department of Neurosurgery, Tianjin Medical University General Hospital, Tianjin, China; ^2^ Laboratory of Neuro-Oncology, Tianjin Medical University General Hospital, Tianjin, China; ^3^ Department of Neurosurgery, Beijing Tsinghua Changgung Hospital, School of Clinical Medicine, Tsinghua Medicine, Tsinghua University, Beijing, China

**Keywords:** FGFR3-TACC3 fusion gene, glioma, malignant progression, invasion, migration, STAT3 signaling pathway

In the published article, there was an error in **Figure 1F** and **Figure 3J** as published. The error in the image presented in **Figure 1F** (U87MG F3-T3 K508R and U87MG Empty vector) and an incorrect use of scale bar in **Figure 3J**. The corrected **Figure 1**, **Figure 3** and their captions appear below.

**Figure 1:**
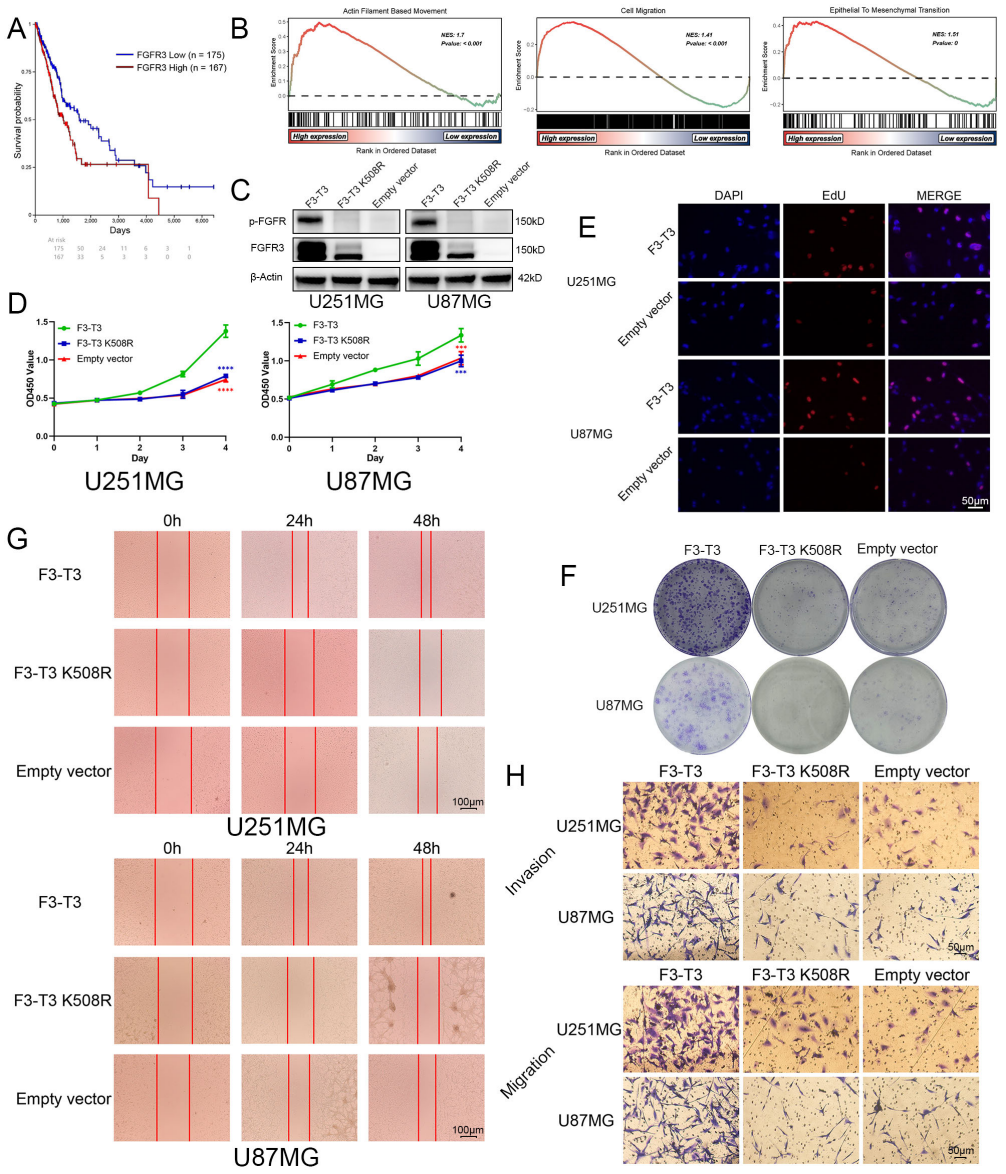
F3-T3 significantly promoted the proliferation, invasion, and migration of glioma cells. **(A)** Correlation of FGFR3 expression with prognosis in glioma patients from the TCGA database: The Kaplan-Meier plotter showed Glioma patients with higher FGFR3 expression reached poorer prognosis than those expressed lower. **(B)** GSEA results of F3-T3 and empty vector based on the GSE42401 database. The GSEA results showed F3-T3 glioma cells enriched in several key signaling pathways that closely correlated with cancer. **(C)** Western blot analysis showing expression levels of p-FGFR and FGFR3 in cells transfected with F3-T3, F3-T3 K508R, and empty vector. **(D)** CCK-8 assay results indicating enhanced proliferation of U87MG and U251MG cells expressing F3-T3 compared to those with F3-T3 K508R mutant and empty vector. **(E)** EdU labeling assay demonstrating increased proliferation in U87MG and U251MG cells due to F3-T3 expression. **(F)** Colony formation assay results showing higher colony formation efficiency in glioma cells expressing F3-T3 compared to control groups. **(G)** Wound healing assay results revealing increased invasion capability in glioma cells with F3-T3 expression compared to cells with F3-T3 K508R mutant and empty vector. (H) Transwell assays for invasion and migration showing that F3-T3 can significantly enhance these abilities in glioma cells compared to F3-T3 K508R and empty vector. (***p<0.001, ****p<0.00001)..

**Figure 3:**
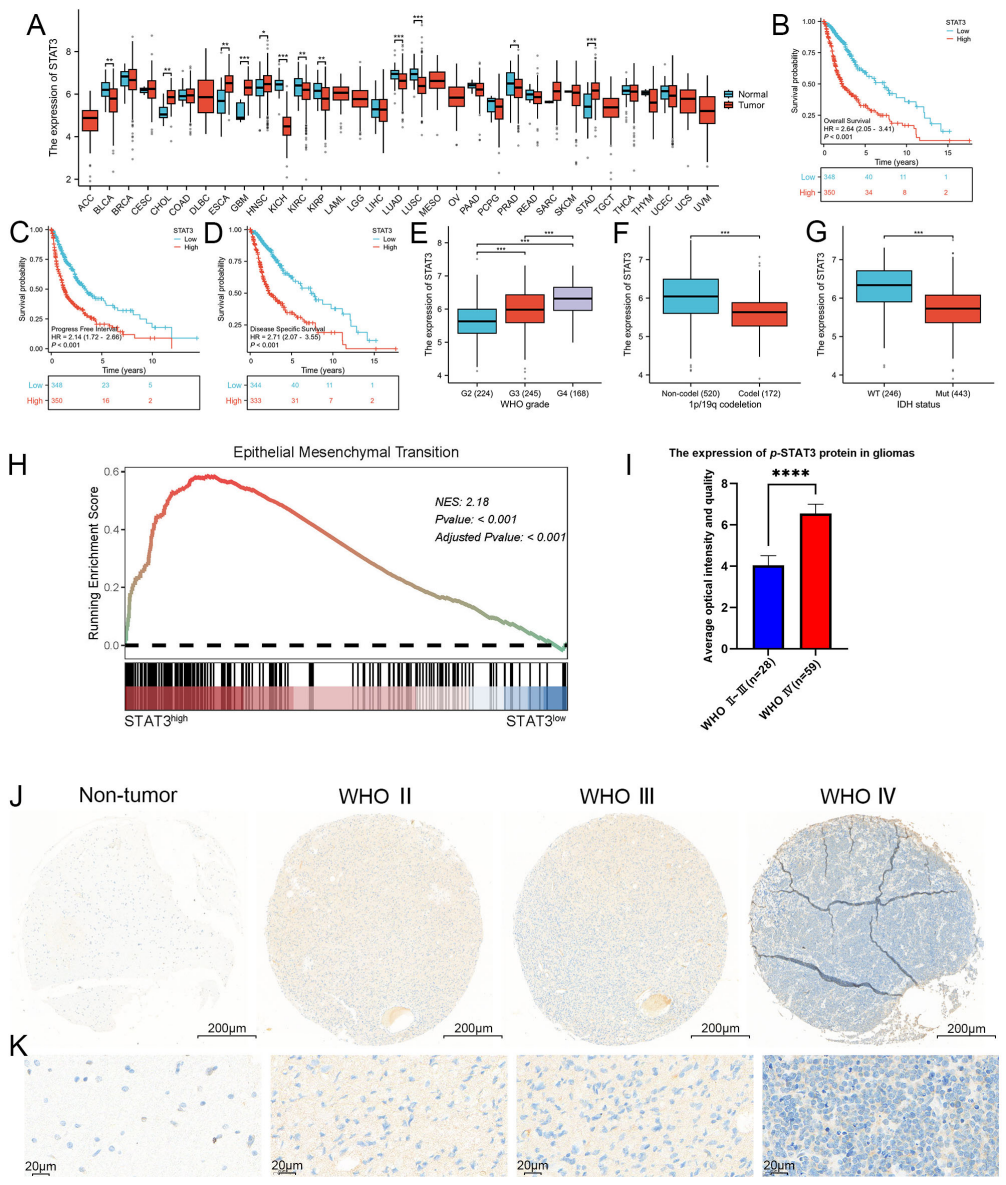
STAT3 was closely associated with the WHO grade, IDH wild-type status, 1p/19q chromosome co-deletion and worse prognosis, as well as being closely linked to EMT. **(A)** Expression profile of STAT3 across 33 different cancer types and their corresponding normal tissues, as shown in the TCGA database. Our analysis revealed that in 9 of the 33 malignant tumor types, the expression profiles did not match those of their corresponding normal tissues, these malignant tumors including lymphoid neoplasm diffuse large B-cell lymphoma (DLBC), adrenocortical carcinoma (AAC), brain lower grade glioma (LGG), mesothelioma (MESO), acute myeloid leukemia (LAML), uveal melanoma (UVM), testicular germ cell tumors (TCGT), ovarian serous cystadenocarcinoma (OV), and uterine carcinosarcoma (UCS). Among the remaining 24 cancer types, 12 exhibited statistically significant differences in STAT3 expression levels between tumor and normal tissues. These cancers included bladder and urothelial carcinoma (BLCA), esophageal carcinoma (ESCA), cholangiocarcinoma (CHOL), glioblastoma (GBM), kidney renal clear cell carcinoma (KIRC), head and neck squamous cell carcinoma (HNSC), kidney chromophobe (KICH), lung adenocarcinoma (LUAD), kidney renal papillary cell carcinoma (KIRP), stomach adenocarcinoma (STAD), lung squamous cell carcinoma (LUSC), and prostate adenocarcinoma (PRAD). In contrast, the other 12 cancers did not show statistically significant differences. Specifically, five cancer types demonstrated elevated STAT3 expression in tumor tissues, including cholangiocarcinoma (CHOL), glioblastoma (GBM), esophageal carcinoma (ESCA), head and neck squamous cell carcinoma (HNSC), and stomach adenocarcinoma (STAD), while seven showed higher expression in normal tissues, including bladder and urothelial carcinoma (BLCA), kidney renal clear cell carcinoma (KIRC), kidney chromophobe (KICH), lung squamous cell carcinoma (LUSC), kidney renal papillary cell carcinoma (KIRP), lung adenocarcinoma (LUAD), and prostate adenocarcinoma (PRAD). Notably, GBM exhibited a marked difference in STAT3 expression between tumor and normal tissues (p < 0.001). **(B–D)** Kaplan-Meier survival curves analyzing the overall survival, progression-free interval, and disease-specific survival of glioma patients categorized by high versus low STAT3 expression. The curves indicate that higher STAT3 expression was associated with poorer prognosis in glioma patients. **(E–G)** Correlation analysis between STAT3 expression levels and clinical parameters in glioma, including WHO grades, 1p/19q chromosome co-deletion status, and IDH mutation status, utilizing the TCGA database. The analysis results shows that STAT3 expression was significantly linked to these clinical indicators. **(H)** GSEA analysis revealed a strong correlation between STAT3 expression and the EMT process, with a NES of 2.1825 and an adjusted p-value < 0.001. **(I, J)** Analysis of p-STAT3 expression in GBM, demonstrating significantly higher levels compared to lower-grade gliomas. **(K)** Enlarged histological image results of (J). (*p<0.05, **p<0.01, ***p<0.001, ****p<0.00001).

The original version of this article has been updated.

