# Supplementary material for: FGFR3-TACC3 fusion gene promotes glioblastoma malignant progression through the activation of STAT3 signaling pathway
Source: Front Oncol. 2025 Apr 8;15:1560008. doi: 10.3389/fonc.2025.1560008 (PMC12011601; doi:10.3389/fonc.2025.1560008)
Supplement: Supplementary file 1 [file DataSheet1.docx]

Supplementary Material

# Supplementary Table S1: STAT3 siRNA sequence

1. Figure S1: Quantized images of colony experiments


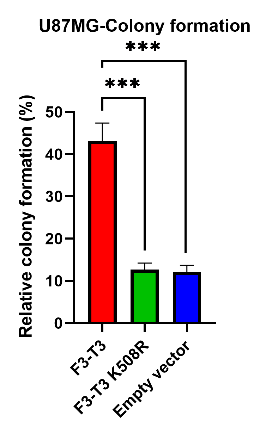

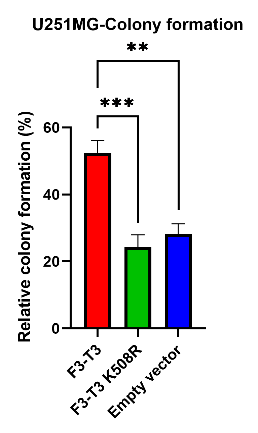


1. Figure S2: Quantized images of wound healing assay


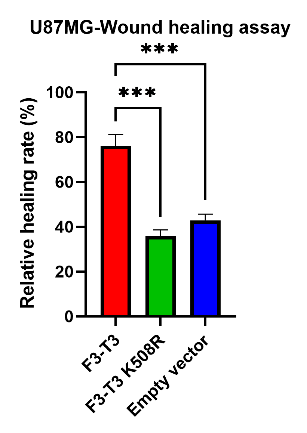

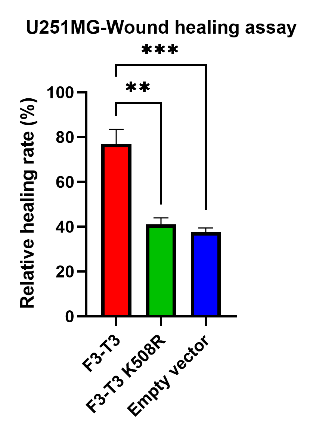


1. Figure S3: Quantized images of transwell assay
2. Transwell invasion assay


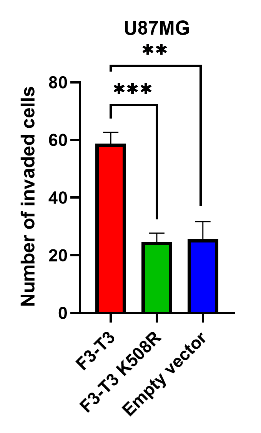

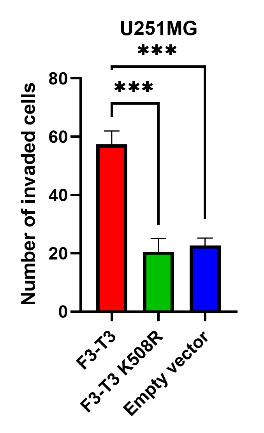


1. Transwell migration assay


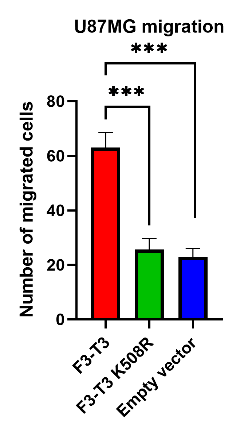

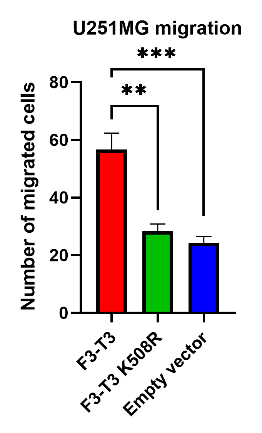


1. Figure S4: Specific figures and statistics of EMT in E-MTAB-6037 gene chip
2. GSEA of F3-T3 vs F3-T3 KD

*
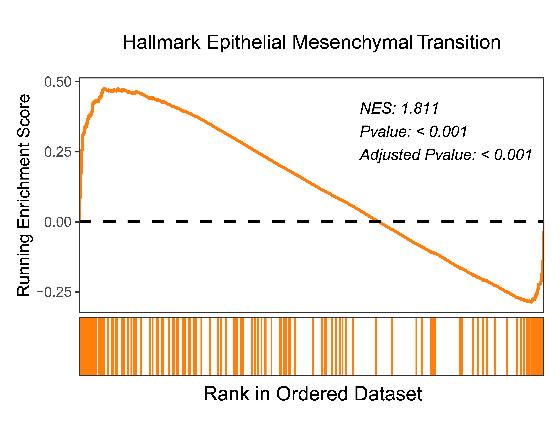
*

1. GSEA of F3-T3 vs F3-T3 PD

*
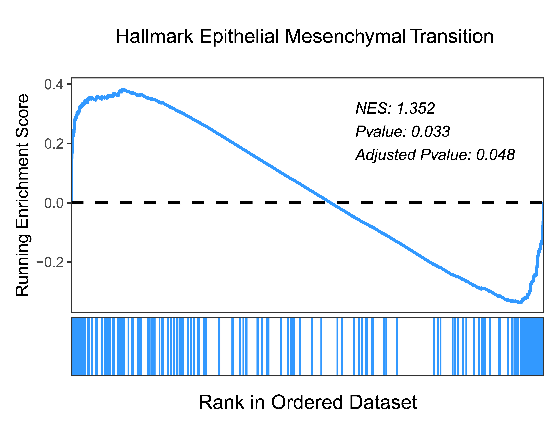
*

1. GSEA of F3-T3 vs Empty vector


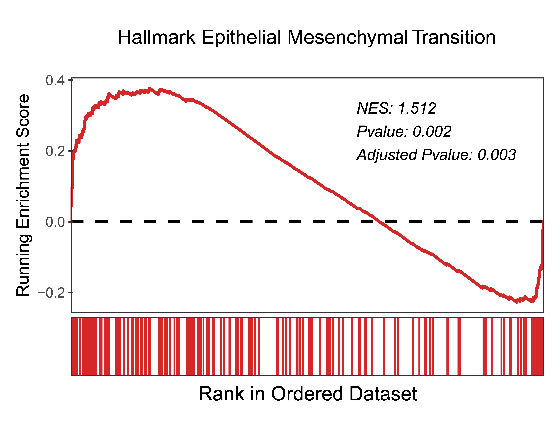


1. Figure S5: IHC results of p-STAT3 in the tissue microarray.


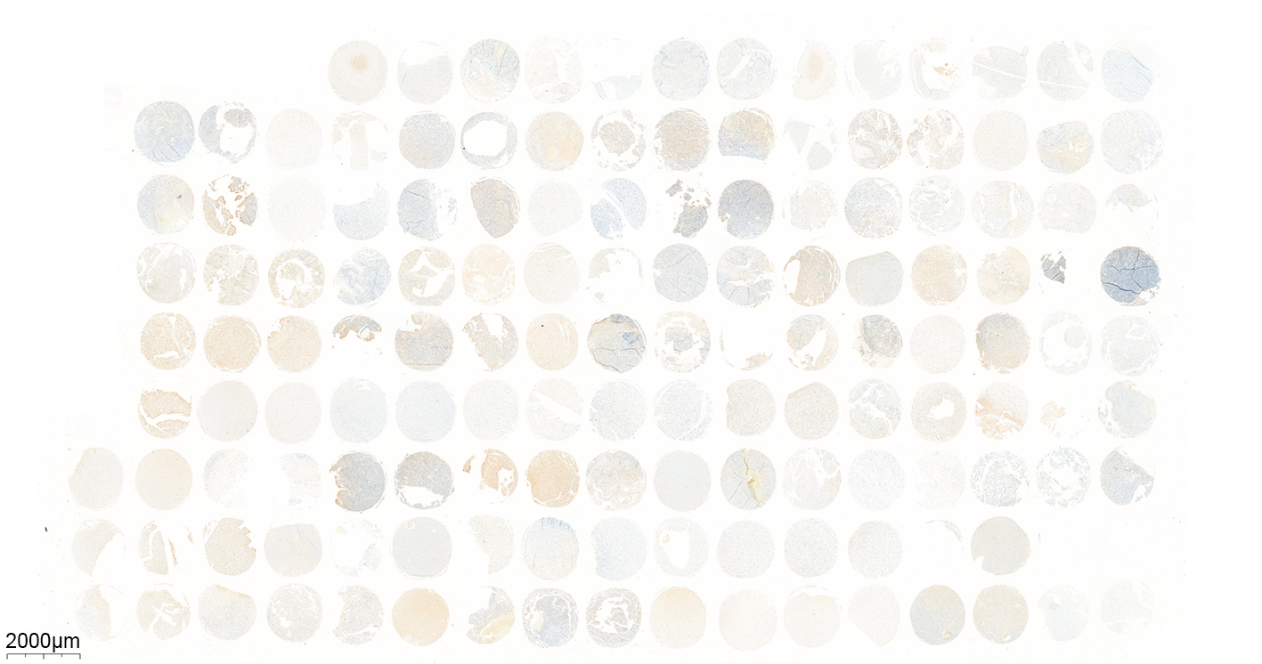


1. Table S1: The top 20 KEGG enrichment pathways and their p-value in RNA sequencing

| ID | Description | GeneRatio | BgRatio | | pvalue | p.adjust | qvalue | Count | |
| --- | --- | --- | --- | --- | --- | --- | --- | --- | --- |
| hsa04657 | IL-17 signaling pathway | 11/308 | 75/7859 | 0.000146 | | 0.005787 | 0.005787 | 11 |  |
| hsa05165 | Human papillomavirus infection | 27/308 | 329/7859 | 0.000207 | | 0.007179 | 0.007179 | 27 |  |
| hsa04928 | Parathyroid hormone synthesis, secretion and action | 12/308 | 100/7859 | 0.000504 | | 0.014185 | 0.014185 | 12 |  |
| hsa04151 | PI3K-Akt signaling pathway | 26/308 | 330/7859 | 0.00051 | | 0.014185 | 0.014185 | 26 |  |
| hsa04061 | Viral protein interaction with cytokine and cytokine receptor | 7/308 | 39/7859 | 0.000692 | | 0.017485 | 0.017485 | 7 |  |
| **hsa04630** | **JAK-STAT signaling pathway** | **13/308** | **124/7859** | **0.001092** | | **0.025309** | **0.025309** | **13** |  |
| hsa04668 | TNF signaling pathway | 12/308 | 121/7859 | 0.0027 | | 0.057729 | 0.057729 | 12 |  |
| hsa04072 | Phospholipase D signaling pathway | 13/308 | 140/7859 | 0.003265 | | 0.061735 | 0.061735 | 13 |  |
| hsa05200 | Pathways in cancer | 31/308 | 478/7859 | 0.003715 | | 0.061735 | 0.061735 | 31 |  |
| hsa04080 | Neuroactive ligand-receptor interaction | 15/308 | 176/7859 | 0.003743 | | 0.061735 | 0.061735 | 15 |  |
| hsa05321 | Inflammatory bowel disease | 6/308 | 39/7859 | 0.003775 | | 0.061735 | 0.061735 | 6 |  |
| hsa05146 | Amoebiasis | 9/308 | 83/7859 | 0.004985 | | 0.076988 | 0.076988 | 9 |  |
| hsa05414 | Dilated cardiomyopathy | 9/308 | 90/7859 | 0.008444 | | 0.123556 | 0.123556 | 9 |  |
| hsa05417 | Lipid and atherosclerosis | 15/308 | 196/7859 | 0.009947 | | 0.138264 | 0.138264 | 15 |  |
| hsa05120 | Epithelial cell signaling in Helicobacter pylori infection | 8/308 | 78/7859 | 0.010997 | | 0.145583 | 0.145583 | 8 |  |
| hsa04727 | GABAergic synapse | 7/308 | 67/7859 | 0.015405 | | 0.194658 | 0.194658 | 7 |  |
| hsa05410 | Hypertrophic cardiomyopathy | 8/308 | 86/7859 | 0.019049 | | 0.214861 | 0.214861 | 8 |  |
| hsa04974 | Protein digestion and absorption | 7/308 | 70/7859 | 0.019237 | | 0.214861 | 0.214861 | 7 |  |
| hsa04610 | Complement and coagulation cascades | 6/308 | 55/7859 | 0.019928 | | 0.214861 | 0.214861 | 6 |  |
| hsa04915 | Estrogen signaling pathway | 10/308 | 121/7859 | 0.020095 | | 0.214861 | 0.214861 | 10 |  |

1. Figure S6: EdU experiments of U251MG after STAT3 was knocked down


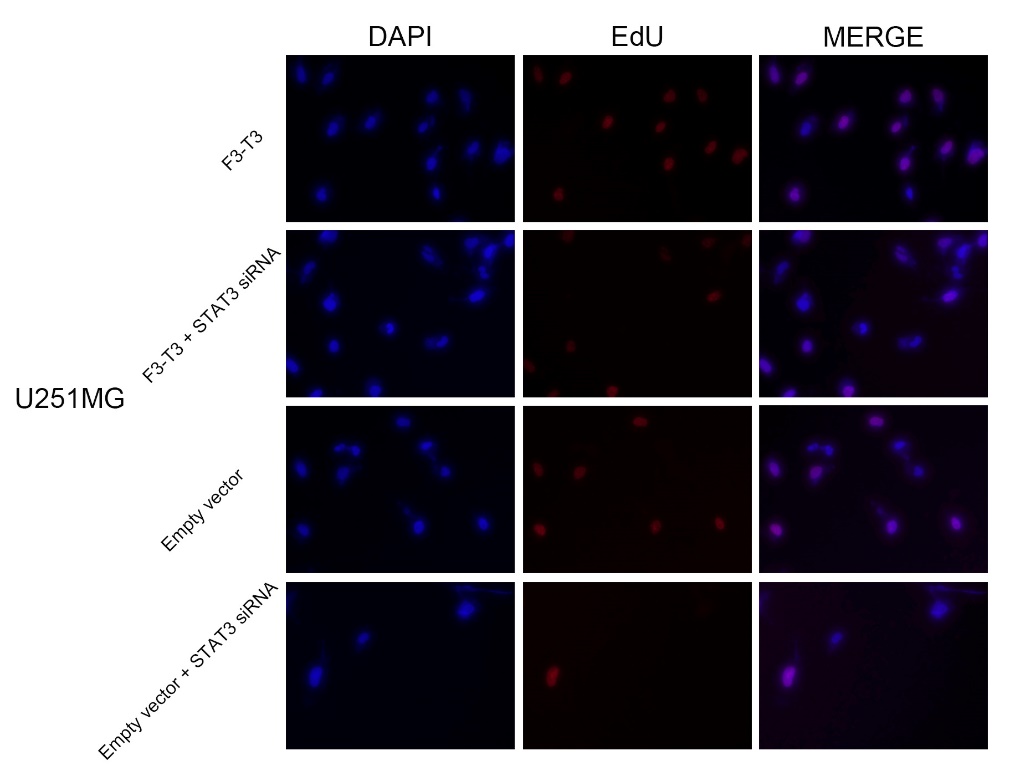


1. Figure S7: Quantized images of wound healing assay after STAT3 was knocked down


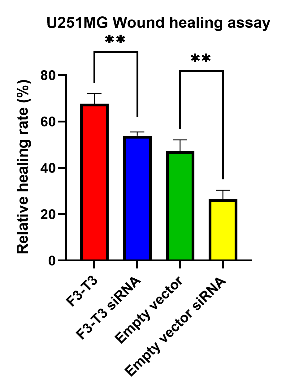

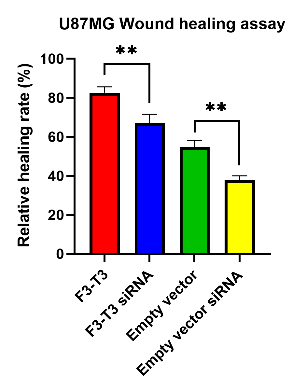


1. Figure S8: Quantized images of transwell experiment after STAT3 was knocked down

Invasion


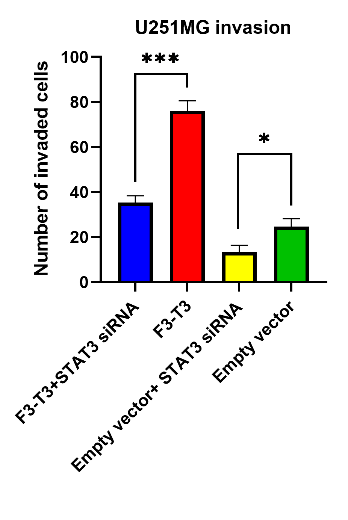

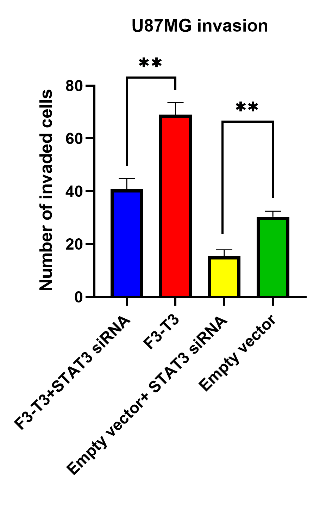


1. Figure S9: Quantized images of transwell experiment after STAT3 was knocked down:

Migration


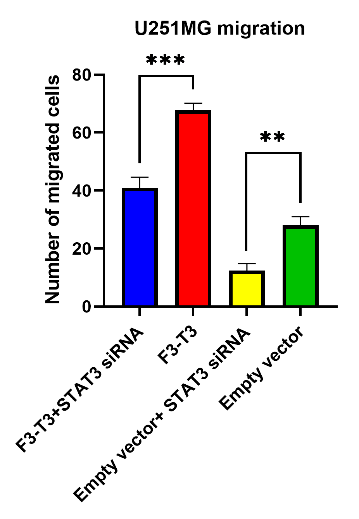

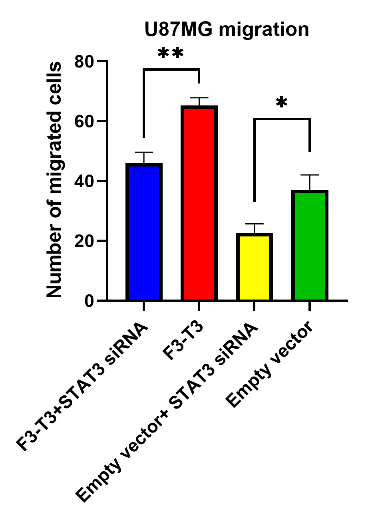


Note: **p*<0.05, ***p*<0.01, ****p*<0.001, *****p*<0.00001
