# Supplementary material for: FGFR3-TACC3 fusion gene promotes glioblastoma malignant progression through the activation of STAT3 signaling pathway
Source: Front Oncol. 2025 Apr 8;15:1560008. doi: 10.3389/fonc.2025.1560008 (PMC12011601; doi:10.3389/fonc.2025.1560008)
Supplement: Supplementary file 2 [file DataSheet2.docx]

Supplementary Methods

1. **Bioinformatic analysis and RNA sequencing**

In E-MTAB-6037 gene chip database, overexpression of F3-T3 protein was induced in human astrocytes, and the global gene expression profiles of these cells were compared with those treated with the specific inhibitor FGFR3-TK (PD173074). Moreover, comparisons were made between human astrocytes containing F3-T3 and those expressing a kinase-inactive variant (F3-T3 K508M) or an empty vector (vec). This data is accessible through the ArrayExpress database under the identifier E-MTAB-6037 (https://www.ebi.ac.uk/biostudies/arrayexpress). The identification of differentially expressed genes (DEGs) was performed using the "DESeq2" package in R, and Gene Set Enrichment Analysis (GSEA) was carried out employing the "clusterProfiler" package.

The GSE42401 database includes the RNA sequencing data from SNB19 and U251MG GBM cell lines transfected with FGFR3-TACC3, FGFR wildtype and TACC3 wildtype (https://www.ncbi.nlm.nih.gov/geo/). In this study, we conducted DEGs between SNB19 GBM cells harboring F3-T3 and those expressing Empty vector. Furthermore, Gene Set Enrichment Analysis (GSEA) was conducted based on the DEGs.

The mRNA-seq data from GTEx and TCGA were downloaded from the UCSC XENA database system (https://xenab rowser.net/datapages/). The STAT3 and FGFR3 gene expression were obtained from the TCGA database (https://cancergenome.nih.gov). Kaplan-Meier (KM) survival analysis was utilized to determine the survival disparities linked to FGFR3 and STAT3 expression in glioma patients. Additionally, STAT3 expression was analyzed in relation to WHO grade, IDH mutation status, and 1p/19q co-deletion using data from the TCGA database.

1. **Cell culture**

Human glioma cell lines U251MG and U87MG were acquired from the American Type Culture Collection (ATCC, US). All glioma cells were maintained in DMEM medium (Gibco, USA) supplemented with 10% fetal bovine serum (FBS, Procell, China). The cells were cultured in an incubator at 37°C containing 5% carbon dioxide (Thermo Scientific).

1. **Lentivirus and siRNA transfection**

Lentiviruses were obtained from Genechem (China) and used to establish the F3-T3 sequences and the F3-T3 kinase-inactive type (F3-T3 K508R). ACTB served as the internal control. The lentiviral transfection followed the protocols provided by the manufacturer. After infection, cells that successfully transfected were enriched using puromycin selection. The efficiency of the transfection was confirmed through western blot analysis. STAT3 siRNA, obtained from Genepharma (China), was transiently introduced into cells using Lipofectamine 3000 (Invitrogen, US).

FGFR3-TACC3 sequence:

- Forward: 5’- GGACCTGGACCGTGTCCTTA-3’,
- reverse: 5’- TTGGAAAGTTCCTTCTGCTTC-3’

FGFR3-TACC3 K508R:

- Forward: 5’-TGCGTCGTGGAGAACAAGTTT-3’,
- reverse: 5’-GCACGGTAACGTAGGGTGTG-3’.

STAT3 siRNA sequence: Exhibited in supplementary materials

STAT3 siRNA sequence

| Gene | Sense (5’-3’) | Antisense (3’-5’) |
| --- | --- | --- |
| STAT3-homo-1839 | GGGACCUGGUGUGAAUUAUTT | AUAAUUCACACCAGGUCCCTT |
| STAT3-homo-1382 | CCCGGAAAUUUAACAUUCUTT | AGAAUGUUAAAUUUCCGGGTT |
| STAT3-homo-2172 | GCUGAACAACAUGUCAUUUTT | AAAUGACAUGUUGUUCAGCTT |
| STAT3-homo-978 | GCAACAGAUUGCCUGCAUUTT | AAUGCAGGCAAUCUGUUGCTT |

1. **Cell Counting Kit-8 (CCK-8) and 5-ethynyl-2'-deoxyuridine (EdU) assays**

The detailed protocol of CCK-8 has been described in previously. Briefly, after 2,000 cells were plated, we measuring the absorbance for four days. Before measuring, the CCK-8 solution was incubated at 37°C for 1 hour. The absorbance of the 96-well plates was then measured at 450 nm to calculate the relative cell proliferation capacity. The main EdU labeling procedure was performed following the instruction book.

1. **Transwell assay**

In the migration assay, glioma cells (1.0×104 cells/well) were seeded in the upper chamber of the insert with serum-free DMEM. The lower chamber contained a medium with 10% FBS to serve as a chemoattractant. After 48 hours of incubation, cells that did not invade through the membrane were removed with a cotton swab from the upper side of the insert. The cells that migrated to the lower side were fixed with 4% paraformaldehyde (P1110, Solarbio, China) and stained with 2.5% crystal violet (G1061, Solarbio, China). For invasion assays, the bottom of the insert was coated with Matrigel, and about 2×104 cells/well were placed in the upper chamber and incubated for 72 hours. The procedures for cell culture and processing in the invasion assay were the same as those in the migration assay. The number of cells that migrated or invaded to the lower surface of the inserts was then quantified.

1. **Colony formation assay**

For the colony formation assays, 1000 glioma cells were evenly seeded in 6-well plates and incubated for two weeks. Following incubation, the cells were washed, fixed, and then stained with 2.5% crystal violet solution. The efficiency of colony formation was subsequently quantified and compared.

1. **Cell wound healing assay**

Approximately 4×105 cells per well were seeded into 6-well cell culture inserts. Once the cells reached 80% confluence, three separate lines were scratched into the cell layer. After scratching, the plates were washed with PBS to remove any detached cells. After serum-free medium was added, initial microscopic images were taken. Additionally, after the glioma cells were incubated for 24 and 48 hours, further images were captured to assess cell migration.

1. **Western blotting**

The main procedure used for western blotting has been described previously. The primary antibodies utilized for the western blots are listed: FGFR3 (Santa Cruz, sc-13121), phosphorylated FGFR (p-FGFR, CST, 3471), STAT3 (CST, 9139), phosphorylated STAT3 (p-STAT3, CST, 9145), N-Cadherin (CST, 13116), Snail (CST, 3879), E-Cadherin (CST, 3195), Vimentin (CST, 5741), and β-Actin (ZSGB-BIO, TA-09). The results were normalized to β-actin levels and analyzed using a relative standard curve.

1. **Tissue microarray**

TMA samples were obtained from clinical specimens of glioma patients who underwent surgical resection at our department from August 2011 and April 2017. Each specimen was pathologically examined and diagnosed by a specialized neuropathologist following the established WHO criteria. Written informed consent was secured from all patients and their families. The TMA comprised 143 samples: 5 were normal tissue, 24 were peritumoral, 24 were borderline, and the remaining 90 were from the central part of the tumors, including 3 WHO grade I, 17 WHO grade II, 11 WHO grade III, and 59 WHO grade IV samples. This study was conducted in accordance with the Helsinki Declaration and received approval from the Ethics Committee of Tianjin Medical University General Hospital.

1. **Xenograft mouse model**

In the current study, U87MG cells, stably transfected with F3-T3, sh-STAT3+F3-T3, or empty vector, were injected into the brains of mice in three separate groups. BALB/c-nu female nude mouse aged 6 weeks (weight approximately 17g) was obtained for vivo experiments. Monitoring began on the 7th day post-implantation, with daily checks of body weight and overall survival (OS), and weekly assessments of tumor burden using the IVIS Spectrum Live Imaging System (Perkin Elmer, USA). Following the death of the mice, their brains were extracted, fixed in formalin, and embedded in paraffin. Subsequent H&E and IHC staining were conducted on these tissue sections. The animal experiments in this study received approval from the Ethical Committee of Tianjin Medical University General Hospital (Ethical approval number: IRB2021-KY-035).

1. **H&E staining, immunohistochemistry (IHC)**

For the immunohistochemistry (IHC) of tissue microarrays (TMA), a p-STAT3 antibody was employed (Cell Signaling Technology, CST, 9145). Additional IHC antibodies used included p-FGFR (Affinity, AF8439), Ki-67 (CST, 9449), and p-STAT3 (CST, 9139). Following incubation with a goat anti-rabbit IgG assay kit (ZSGB-Bio, China), IHC markers were visualized. The IHC and H&E staining images were captured using a VANOX microscope (Olympus).

1. **Statistical analysis**

All experiments in this study were replicated a minimum of three times. Statistical analyses were performed using SPSS software (version 22). Quantitative data are expressed as the mean ± standard deviation (SD). Survival analyses were conducted using the log-rank test through Graphpad Prism (version 9.0). Spearman’s correlation coefficient was utilized for correlation analyses. Comparisons between two groups were made using an unpaired t-test, with a p-value of less than 0.05 considered statistically significant.
